# Hollow CoP/FeP_4_ Heterostructural Nanorods Interwoven by CNT as a Highly Efficient Electrocatalyst for Oxygen Evolution Reactions

**DOI:** 10.3390/nano11061450

**Published:** 2021-05-30

**Authors:** Yanfang Liu, Yong Li, Qi Wu, Zhe Su, Bin Wang, Yuanfu Chen, Shifeng Wang

**Affiliations:** 1 College of Science, Institute of Oxygen Supply, Tibet University, Lhasa 850000, China; liuyanfang@utibet.edu.cn (Y.L.); xzuliyong@utibet.edu.cn (Y.L.); wuqi_zangda@163.com (Q.W.); 2 School of Electronic Science and Engineering, and State Key Laboratory of Electronic Thin Films and Integrated Devices, University of Electronic Science and Technology of China, Chengdu 610054, China; su_zhe0412@hotmail.com; 3 Key Laboratory of Cosmic Rays, Tibet University, Ministry of Education, Lhasa 850000, China

**Keywords:** CoP-FeP_4_ heterojunction, hollow nanorods, oxygen evolution reaction

## Abstract

Electrolysis of water to produce hydrogen is crucial for developing sustainable clean energy and protecting the environment. However, because of the multi-electron transfer in the oxygen evolution reaction (OER) process, the kinetics of the reaction is seriously hindered. To address this issue, we designed and synthesized hollow CoP/FeP_4_ heterostructural nanorods interwoven by carbon nanotubes (CoP/FeP_4_@CNT) via a hydrothermal reaction and a phosphorization process. The CoP/FeP_4_@CNT hybrid catalyst delivers prominent OER electrochemical performances: it displays a substantially smaller Tafel slope of 48.0 mV dec^−1^ and a lower overpotential of 301 mV at 10 mA cm^−2^, compared with an RuO_2_ commercial catalyst; it also shows good stability over 20 h. The outstanding OER property is mainly attributed to the synergistic coupling between its unique CNT-interwoven hollow nanorod structure and the CoP/FeP_4_ heterojunction, which can not only guarantee high conductivity and rich active sites, but also greatly facilitate the electron transfer, ion diffusion, and O_2_ gas release and significantly enhance its electrocatalytic activity. This work offers a facile method to develop transition metal-based phosphide heterostructure electrocatalysts with a unique hierarchical nanostructure for high performance water oxidation.

## 1. Introduction

As a kind of sustainable and clean energy, hydrogen has great application prospects for promoting the commercialization of fuel cells. At present, splitting water is a promising hydrogen generation technology that can not only alleviate the consumption of many fossil fuels, but also protect the environment [1,2]. In the process of water electrolysis, the oxygen evolution reaction (OER) at the anode determines the work efficiency of the whole process. Nevertheless, the process of OER involves multiple electron transfer steps so that it has sluggish reaction dynamics [3,4,5,6]. Although RuO_2_ and IrO_2_ have been considered as good commercial catalysts for OER, the large-scale application of RuO_2_ and IrO_2_ for OER is limited by the resource scarcity and high cost [7,8,9]. Consequently, it is becoming hugely important to develop OER catalysts with low cost, high efficiency and excellent long-term stability [10].

Many researchers have made great efforts to explore the non-noble metal catalysts, such as transition metal hydroxides (e.g., CoFe(OH)_x_ [11]), oxides (e.g., CoO_x_ [12]), phosphides (e.g., NiFeP [13]), selenides (e.g., WSe_2_ [14]), sulfides (e.g., Co_8_FeS_8_ [15]), nitrides (e.g., CoFe-N [16]) and carbides (e.g., Mo_2_C [17]) [18,19,20]. Among all the catalyst types, transition metal phosphides (TMPs) have become the research focus due to their high activity and low cost. However, the electrochemical properties of pure transition metal phosphides are far from as expected. As a result, many researchers have begun to improve the performance of OER in various aspects, such as manipulating the morphology of materials, creating defects, forming heterojunctions and doping extrinsic atoms. At the same time, some bimetallic layered hydroxides also exhibit good stability and expose a large number of active sites due to their unique layered structure. However, the strategies mentioned above are not efficient to improve the electrical conductivity and enlarge the electrochemically active areas. In order to address such issues, introducing highly conductive carbon nanotubes (CNTs) or graphene into the catalyst during the synthesis process have been widely considered [21,22].

Great progress on improving the catalytic activity has been made. For example, Yue et al. [11] first synthesized Cu_2_O micropolyhedra as the sacrificial template using a coprecipitation method, and then synthesized CoFe(OH)_x_ hollow microspheres at room temperature, showing strong stability and excellent electrochemical activity for OER with an overpotential of 293 mV at 10 mA cm^−2^ and a Tafel slope of 67.4 mV dec^−1^, respectively. Zhou et al. [23] synthesized two-dimensional bimetalline CoFe selenide using a simple metal ion self-assembly method under hydrothermal conditions, with an overpotential of 257 mV and a low Tafel slope of 45.6 mV dec^−1^ at 10 mA cm^−2^. The outstanding electrochemical activity of OER is attributed to the unique two-dimensional layered structure of the catalysts, which are conducive to charge transfer and ion diffusion. Liu et al. [24] synthesized CoFe bimetallic layered hydroxide composite with N-doped carbon nanotubes, which showed durable stability. The introduction of CNT increased the specific surface area of electrochemical activity and conductivity as well. The preparation process was carried out by a simple one-pot method at room temperature, generating an overpotential of 270 mV at 10 mA cm^−2^, and a Tafel slope of 56.88 mV dec^−1^. However, it is still difficult to develop a highly efficient and stable CNT-composited bimetallic compound OER catalyst with unique nanoarchitecture and abundant catalytic sites.

To address these issues, we designed and synthesized hollow CoP/FeP_4_ heterostructural nanorods interwoven by carbon nanotubes (CoP/FeP_4_@CNT) via a hydrothermal reaction and subsequent phosphorization. This hollow heterostructure hybrid catalyst delivers prominent OER electrochemical performances: it displays an extremely smaller Tafel slope of 48.0 mV dec^−1^ and a lower overpotential of 301 mV at 10 mA cm^−2^, compared with a RuO_2_ commercial catalyst; it also shows good stability over 20 h. The excellent OER performance is mainly due to its unique hollow nanorod structure interwoven with conductive CNT, which guarantees high conductivity and enriches active sites. These merits greatly facilitate the electron transfer, ion diffusion, and O_2_ gas release, which significantly enhance its electrocatalytic activity. This work provides a promising strategy for the synthesis of a hollow transition-metal phosphide heterostructure with an efficient OER performance.

## 2. Materials and Methods

### 2.1. Materials

Cobalt nitrate hexahydrate (Co(NO_3_)_2_·6H_2_O), iron(III) nitrate nonahydrate (Fe(NO_3_)_3_ 9H_2_O), carbon nanotube paste (5% CNT in NMP), and ethanol were purchased from Aladdin, Co. Ltd. (Seoul, Korea). Ruthenium oxide (RuO_2_ electrocatalyst), and Nafion solution were received from shanghai Macklin Ltd. All chemical reagents were analytical grade and used without further purification; double distilled water/ethanol mixtures were used as washing solvents throughout the investigations.

### 2.2. Synthesis of CoFe-LDH@CNT

CoFe-LDH@CNT was synthesized according to the method of the previous report with a slight change [1]. Firstly, 1.2 g of CNT slurry was taken and dispersed into 60 mL of deionized water for an ultrasound for 30 min until the uniform black suspension was formed. Secondly, 404 mg of Fe(NO_3_)_3_·9H_2_O, 292 mg of Co(NO_3_)_2_·6H_2_O, 1.21 g of CO(NH_2_)_2_ and 297 mg of NH_4_F were poured into the above suspension and magnetically stirred for an additional 30 min. Finally, the solution was transferred into a stainless steel autoclave, heated to 120 °C for 12 h. After cooling down naturally, the sample was collected using suction filtration, washed with deionized water and ethanol several times, and then placed in a freeze-dryer to dry overnight. The dried product is lump-like and needs to be ground into powder with a mortar for subsequent testing and characterizations. For comparison, the preparation process for CoFe-LDH and CoFe-LDH@CNT was similar apart from the addition of CNT slurry.

### 2.3. Synthesis of CoP/FeP_4_@CNT nanorods

CoFe-LDH@CNT and NaH_2_PO_2_ with a weight ratio of 1:10 were put on two separate quartz boats with NaH_2_PO_2_ powder at the upstream side of the tube furnace. The phosphorization process was carried out in argon, and the temperature was raised to 400 °C for 2 h at a heating rate of 3 °C min^−1^. When the temperature in the furnace was cooled to room temperature, the resulting sample was recorded as CoP/FeP_4_@CNT. Using CoFe-LDH as the precursor, we synthesized the CoFeP sample.

### 2.4. Characterizations

The crystal structure of the as-obtained CoP/FeP_4_@CNT was analyzed through X-ray diffraction (Palmer naco X’Pert PRO). The Raman spectrum of CoP/FeP_4_@CNT was conducted with a 532 nm excitation laser (Renishaw). The surface chemical composition and electronic structure of CoP/FeP_4_@CNT were examined by an X-ray photoelectron spectroscope on a Thermo Scientific K-Alpha. The morphology of the sample was observed using a United States FEI Inspect F50 Scanning eletronmicroscope (SEM) and an FEI Talos F200x (TEM) equipped with an energy dispersive X-ray detector (EDX, Brooke quantax).

### 2.5. Electrocatalytic Measurements

All electrochemical performance tests in the OER process were performed on an electrochemical workstation (CHI660D) with a three-electrode system, and 1 M KOH solution was used as the electrolyte. Generally, the graphite rod was used as the counter electrode, Hg/HgO electrode as the reference electrode, and the glassy carbon electrode as the working electrode. In all measurements, potential values were changed to a reversible hydrogen electrode (RHE): E_RHE_ = E_Hg/HgO_ + 0.923 V. Before the electrocatalytic test, the scan of at least 20 cycles of cyclic voltammetry (CV) was performed at 100 mV s^−1^ to ensure to obtain stable curves. Line scan voltammogram (LSV) curves have a potential range of 0 to 0.8 V at a scanning rate of 5 mv s^−1^. The double-layer capacitance (Cdl) was also calculated from cyclic voltammogram (CV) data in 1 M KOH solution, which require sweeping rates from 20 mV s^−1^ to 200 mV s^−1^ over a range of potential tests, with a step of 20 mV s^−1^ for each curve. The electrochemical impedance spectroscopy (EIS) tests were performed from 100 kHz to 0.1 Hz. The stability of CoP/FeP_4_@CNT was evaluated by continuous chronoamperometry using nickel foam (1 × 1 cm), and the holding potential for the chronoamperometry test was 1.533 V.

## 3. Results

**Figure 1:**
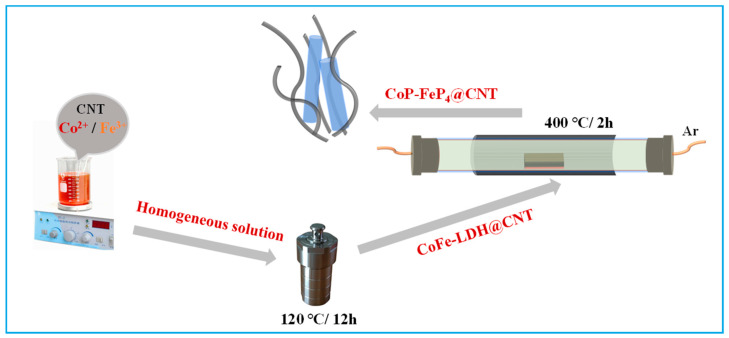
A two-step fabrication process of CoP/FeP_4_@CNT nanorods.

The schematic diagram of CoP/FeP_4_@CNT preparation is clearly presented in Figure 1. Firstly, the CoFe-LDH@CNT nanorod precursor was synthesized by the hydrothermal method, and then the material was further modified by phosphorization. Importantly, the subsequent experimental data showed that the catalytic performance of the catalyst was significantly improved after phosphorization, which was ascribed to the synergistic effects of different phases.

**Figure 2:**
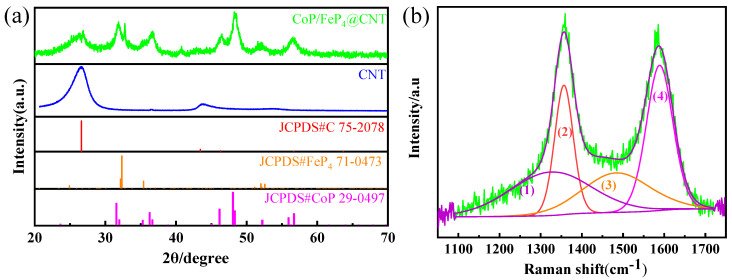
(**a**) The XRD pattern of the CoP/FeP_4_@CNT and CNT, (**b**) Raman spectrum of the CoP/FeP_4_@CNT.

The X-ray diffraction (XRD) pattern (Figure 2a) demonstrates that the characteristic peaks of CoP/FeP_4_@CNT match well with the standard patterns of CoP (PDF no. 29-0497) and FeP_4_ (PDF no. 71-0473). Moreover, the peaks at 26.6° and 43.4° correspond to (111) and (010) of CNT (PDF No. 75-0473). The peaks at 32.6°, 36.7° and 52.2° are (131), (112), and (133) planes of FeP_4;_ the peaks at 31.9°, 36.7°, 46.5°, 48.4°, 52.2° and 56.7° correspond to (011), (102), (112), (202), (103), and (301) planes of CoP. In addition, as shown in Figure S1, the prominent peaks of the CoFe-LDH@CNT precursor correspond perfectly to FeO(OH) (PDF No. 34-1266) and CoOOH (PDF No. 26-0480). The Raman spectrum shows two obvious peaks at 1347 cm^−1^ and 1582 cm^−1^, respectively, corresponding to the D peak and G peak of CNT, as displayed in Figure 2b. The D peak associates with the defect of the carbon crystal, and the G peak demonstrates the in-plane stretching vibration of the sp^2^ hybridization of carbon. The band intensity ratio (I_D_/I_G_) of the CoP/FeP_4_@CNT nanorods was 1.11, suggesting the formation of lattice defects during the phosphidation process. Furthermore, the Raman spectrum was deconvoluted into four peaks, according to previous research [25]. The peaks at 1325 cm^−1^ (1) and 1485 cm^−1^ (3) are related to sp^3^-type carbon, and the other two spectra at around 1357.3 cm^−1^ (2) and 1590.3 cm^−1^ (4) are associated with sp^2^-type carbon. The integrated area ratio of sp^3^ to sp^2^ (A_sp3_/A_sp2_) is 0.98, inferring that the amount of sp^3^-type carbon is nearly the same as the sp^2^-type carbon. Importantly, the catalytic activity of the metal phosphides is related to the surface properties of the material on the one hand and the internal structure on the other hand. Therefore, the electrochemical performance can be improved by increasing the number of exposed active sites, as well as adjusting the morphology, defects and active surface area of the material.

**Figure 3:**
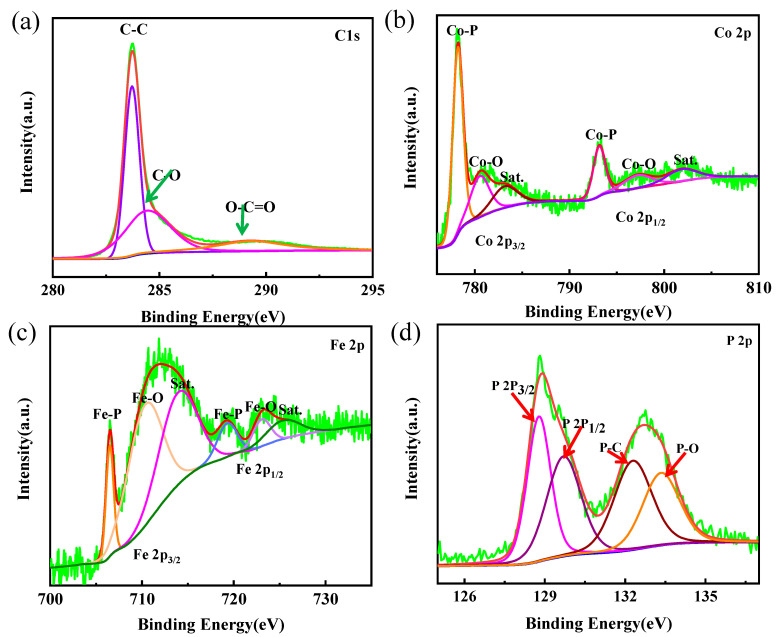
(**a**–**d**) The XPS spectra of (**a**) C 1s, (**b**) Co 2p, (**c**) Fe 2p and (**d**) P 2p of CoP/FeP_4_@CNT nanorods.

X-ray photoelectron spectroscopy (XPS) was used to analyze the type of elements contained in CoP/FeP_4_@CNT and the chemical valence states presented in various elements. As shown in Figure S2, the XPS survey spectrum proves the existence of C, O, Co, Fe, P. Meanwhile, Figure 3 also shows the high-resolution XPS patterns of C, Co, Fe and P, respectively. There are three distinct characteristic peaks in the XPS of C1s, which are located at 283.7, 284.5 and 289.3 eV corresponding to C–C, C–O and O–C=O, respectively (Figure 3a). The Co 2p XPS spectrum shows two peaks at 778.2 eV (Co 2p3/2) and 793.2 eV (Co 2p1/2) which are assigned to the Co–P bond, and there are another two satellites (Sat.) at 783.3 and 802.2 eV. The other peaks at 780.7 and 797.1eV correspond to the Co–O band, which might be due to the sample being oxidized in air (Figure 3b). As shown in Figure 3c, there are two peaks at 706.5 eV (Fe 2p3/2) and 719.5 eV (Fe 2p1/2) in the XPS spectrum of Fe 2p, indicating the presence of a Fe–P bond. The obvious peaks at 710.6 and 723.2 eV, which can be considered as the oxidation state of Fe, and the satellite peaks at 714.1 and 725.6 eV are generated by the oscillation of excited electrons. In Figure 3d, the XPS spectrum of P 2p can be deconvoluted into four distinct peaks at 128.8, 129.7, 132.3 and 133.3 eV corresponding to P 2p_3/2_, P 2p_1/2_, P–C, and P–O, respectively, which represent different chemical states of element P [26,27,28,29,30].

**Figure 4:**
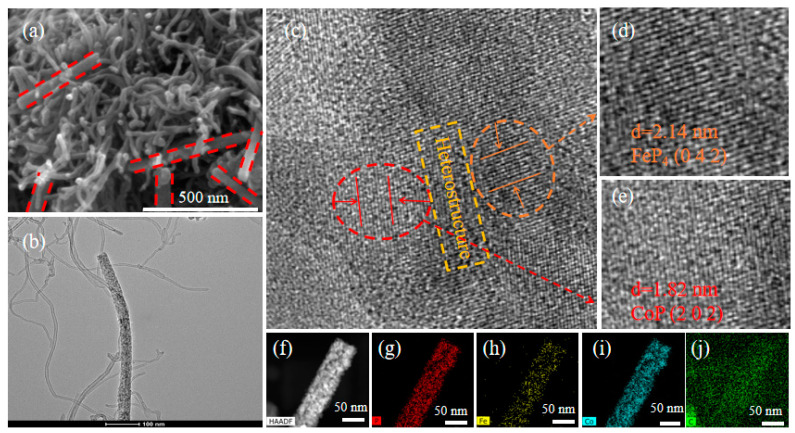
(**a**,**b**) SEM and TEM, (**c**) HRTEM, (**d**,**e**) are magnifications of the orange and red areas in (**c**), respectively, and the value of d in the diagram (**d**,**e**) represents the width of the 10 lattice stripes. (**f**) HAADF-STEM and (**g**–**j**) elemental mapping of CoP/FeP_4_@CNT.

The scanning electron microscopy (SEM) images show that both morphologies of CoFe-LDH and CoFe-LDH@CNT precursors are nanorods (Figures S3 and S4). At the same time, it can be observed that CoFe-LDH nanorods are mostly slender, while CoFe-LDH@CNT nanorods are short and thick. Strangely, almost all the nanorods showed a hollow structure after CoFe-LDH phosphorization, and only a small amount of nanorods contained nanoparticles, indicating that the phosphorization process can play a certain etching role. As shown in Figure 4a, the CoP/FeP_4_@CNT nanorods are clearly obtained after phosphorization for CoFe-LDH@CNT, and it is also found that the original morphology does not change after phosphorization. The unique difference is the formation of hollow nanorods after phosphorization, which could well explain the significant enhancement in the electrochemical performance after phosphorization. The hollow nanorods provide a channel for electron transfer, which is beneficial to speed up the reaction.

In Figure 4b, the transmission electron microscopy (TEM) image clearly presents that the diameter of CoP/FeP_4_@CNT hollow nanorods is about 40 nm. The crystal lattice fringes in the orange circle region in Figure 4c corresponds to the (0 4 2) crystal plane of FeP_4_ (Figure 4d), and lattice fringes in the red circle region in Figure 4e corresponds to the (2 0 2) crystal plane of CoP; these results are consistent with XRD data (Figure 2a) [31,32]. It is worth noting that the middle yellow rectangular area depicts the formation of heterojunctions during phosphorization. Therefore, another reason for the excellent catalytic properties after phosphorization can be put down to the enhancement effect between the phases of the two metal phosphides. In addition, the high-angle annular dark-field scanning TEM (HAADF-STEM) image (Figure 4f) and the element mapping images (Figure 4g–j) show that the C, Co, Fe and P are uniformly distributed in the material. Meanwhile, this information can also be further verified by the EDX data (Figure S5).

**Figure 5:**
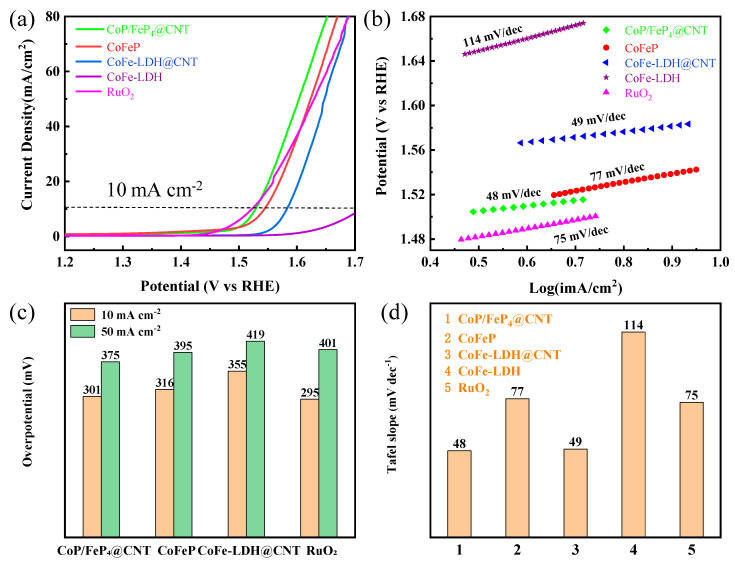
(**a**) LSV curves in 1 M KOH solution, (**b**) Tafel plots, (**c**) the potential of 10 mA cm^−2^ and 50 mA cm^−2^, (**d**) the bar graph of the Tafel plots.

The electrochemical tests of all samples were conducted in a 1 M KOH solution with a glassy carbon electrode as the working electrode. In Figure 5a, the CoFe-LDH precursor without CNT showed the worst catalytic activity, with an overpotential of 485 mV (at 10 mA cm^−2^) and an extremely high Tafel slope (114 mV dec^−1^). In contrast, the precursor CoFe-LDH@CNT exhibits excellent catalytic activity with an overpotential of 355 mV and a relatively low Tafel slope of only 49 mV dec^−1^. Therefore, the better catalytic activity of the CoFe-LDH@CNT is ascribed to the high conductivity and a large electrochemical activity specific surface area provided by the CNT networks. Next, the differences between the electrochemical properties of the precursor (CoFe-LDH and CoFe-LDH@CNT) and the phosphide (CoFeP and CoP/FeP_4_@CNT) were compared. Similarly, the compounds with CNT performed better than those without CNT. Another important finding was that both the required overpotential and the Tafel slope of the phosphide at 10 mA cm^−2^ performed better than those of the precursors. Therefore, it is safe to conclude that the catalytic activity of the material can be enhanced through the phosphorization process.

In Figure 5c, CoP/FeP_4_@CNT displays a lower overpotential of 301 mV compared with other samples at the current density of 10 mA cm^−2^. Meanwhile, CoP/FeP_4_@CNT exhibits the optimal Tafel slope (48 mV dec^−1^) among all the samples. Although the as-prepared CoP/FeP_4_@CNT demonstrates a lager overpotential than that of the commercial RuO_2_ at 10 mA cm^−2^, it surpasses RuO_2_ when the current density increases. For example, at the current density of 50 mA cm^−2^, the potential of CoP/FeP_4_@CNT is only 375 mV, while that of RuO_2_ is 401 mV and CoP/FeP_4_@CNT behaves best at a high current density, as shown in Figure 5c. By comparing the Tafel slopes of all the tested samples, the CoP/FeP_4_@CNT is the minimum, which is anastomotic with the results of the LSV. Therefore, an important conclusion can be drawn that CoP/FeP_4_@CNT possesses a better dynamic performance for the OER.

**Figure 6:**
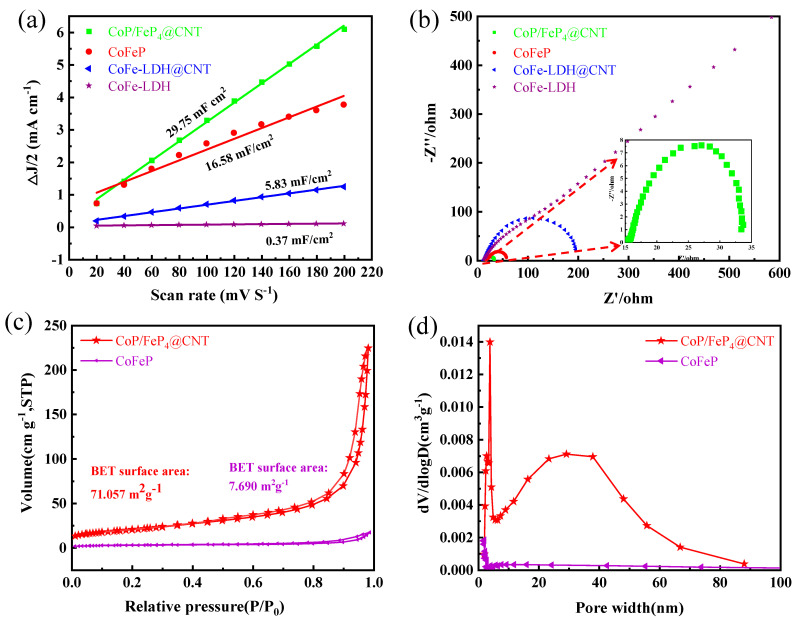
(**a**) Estimated C_dl_ values and relative electrochemically active surface areas, and (**b**) Nyquist plots of CoP/FeP_4_@CNT, CoFeP, CoFe-LDH@CNT and CoFe-LDH. The inset of (**b**) is the magnification of the EIS spectrum of CoP/FeP_4_@CNT. (**c**) N_2_ adsorption/desorption isotherms, and (**d**) pore-size distribution plots of CoFeP and CoP/FeP_4_@CNT.

In order to fully understand the origin of the outstanding electrochemical performance of CoP/FeP_4_@CNT, the electrochemical double-layer capacitances (C_dl_) were examined by operating cyclic voltammograms (CVs) in a current range, which can directly estimate the electrochemical active surface area (ECSA). Specifically, CVs with different scan rates (20–200 mV s^−1^) are demonstrated in a potential range between 1.203 and 1.303 V (vs. RHE). As shown in Figure 6a, the C_dl_ of CoP/FeP_4_@CNT (29.75 mF cm^−2^) is higher than that of CoFeP (16.58 mF cm^−2^), CoFe-LDH@CNT (5.83 mF cm^−2^) and CoFe-LDH (0.37 mF cm^−2^). The large C_dl_ of CoP/FeP_4_@CNT indicates a substantially large active surface area, which is beneficial for charge transfer and ion diffusion. In Figure S6, the CVs of all samples were shown at the different scan rates. In addition, CoP/FeP_4_@CNT displays the smallest semicircle diameter in electrochemical impedance spectroscopy (EIS) in Figure 6b, which indicated that CoP/FeP_4_@CNT possesses the minimum electrochemical impedance and the fastest charge transfer rate [33]. In order to get the aperture information for the CoP/FeP_4_@CNT, the BET test was acquired through N_2_ adsorption–desorption–isotherm in Figure 6c. The CoP/FeP_4_@CNT shows a BET surface area of 71.057 m^2^ g^−1^, which is ten times that of CoFeP (7.690 m^2^ g^−1^) [34,35,36]. Additionally, the distribution of pore size obtained from the Barrett–Joyner–Halenda (BJH) method is presented in Figure 6d. The CoP/FeP_4_@CNT shows that the pore size has a great probability at the range of 2–5 nm and 20–40 nm, confirming the existence of micropores and mesopores. However, CoFeP mainly exists in the form of micropores, which can perfectly explain why CoP/FeP_4_@CNT has more of an outstanding performance than CoFeP: the large pores make it easier for electrons to pass through and speed up the reaction.

**Figure 7:**
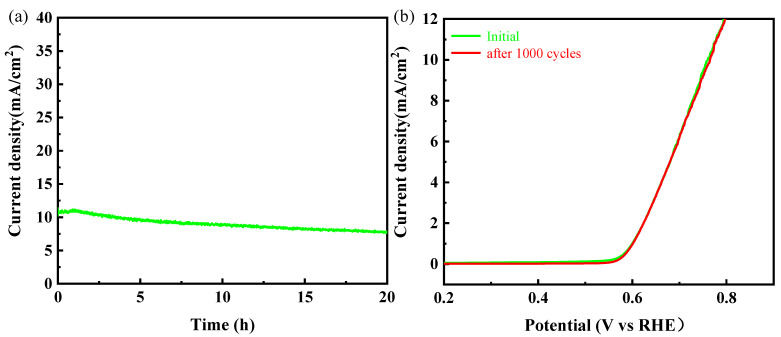
(**a**) *i*~*t* curve of CoP/FeP_4_@CNT with the current density of 10 mA cm^−2^ in 1 M KOH solution at 1.533 V. (**b**) LSV curves of CoP/FeP_4_@CNT before and after 1000 cycles.

In addition to the electrochemical characterization methods described above, the long-term stability of materials is often used as a benchmark to judge the performance of the OER. As shown in Figure 7a, there is no obvious change at 10 mA cm^−2^ after 18 h tests. Furthermore, it was found that CoP/FeP_4_@CNT could still coincide well with the initial one after 1000 cycles, which indicated that CoP/FeP_4_@CNT has excellent long-term stability and has a great prospect for future applications of the OER.

## 4. Conclusions

In summary, hollow CoP/FeP_4_ heterostructural nanorods interwoven by carbon nanotubes were synthesized by a facile hydrothermal reaction and a subsequent phosphorization process. The CoP/FeP_4_@CNT hybrid shows excellent OER performance with a low overpotential of 301 mV to deliver 10 mA cm^−2^ and an extremely low Tafel slope of 48 mV dec^−1^. The remarkable catalytic properties are attributed to its unique nanoarchitecture: the hollow structure of CoP/FeP_4_ nanorods interwoven by CNT. Furthermore, the interface between CoP and FeP_4_ guarantee abundant active sites and a large electrochemical active specific surface area to improve the OER activity; the CoP/FeP_4_ heterostructure induced electronic modulation, and the highly conductive CNT skeleton facilitate the electron transfer thus enhances OER performance. This work provides a strategy to rationally design and facilely fabricate a bimetallic phosphide heterojunction with unique nanoporous architecture with an enhanced OER performance.

## Data Availability

Data is available on the request from the corresponding author.

## Supplementary Materials

The following are available online at https://www.mdpi.com/article/10.3390/nano11061450/s1, Figure S1. XRD pattern of the CoFe-LDH@CNT precursor, Figure S2. The XPS survey spectrum of CoP/FeP_4_@CNT, Figure S3. SEM morphology images of (a) CoFe-LDH, (b) CoFeP, Figure S4. SEM images of CoFe-LDH @CNT from different magnifications, Figure S5. EDX spectrum of CoP/FeP_4_@CNT, Figure S6. Voltammograms for the OER in an alkaline electrolyte.
