# Supplementary material for: Hollow CoP/FeP4 Heterostructural Nanorods Interwoven by CNT as a Highly Efficient Electrocatalyst for Oxygen Evolution Reactions
Source: Nanomaterials (Basel). 2021 May 30;11(6):1450. doi: 10.3390/nano11061450 (PMC8227064; doi:10.3390/nano11061450)
Supplement: Supplementary file 1 [file nanomaterials-11-01450-s001.zip › nanomaterials-1222652-supplementary.pdf]

nanomaterials

Supplementary Materials

Hollow CoP/FeP4 Heterostructural Nanorods Interwoven by CNT as a Highly Efficient Electrocatalyst for Oxygen Evolution Reactions

Yanfang Liu [1,2,†], Yong Li [1,†], Qi Wu [1], Zhe Su [2], Bin Wang [2,*], Yuanfu Chen [1,2,*] and Shifeng Wang [1,3,*]

1 College of Science, Institute of Oxygen Supply, Tibet University, Lhasa 850000, China; liuyanfang@utibet.edu.cn (Y.L.); xzuliyong@utibet.edu.cn (Y.L.); wuqi_zangda@163.com (Q.W.)
2 School of Electronic Science and Engineering, and State Key Laboratory of Electronic Thin Films and Integrated Devices, University of Electronic Science and Technology of China, Chengdu 610054, China; su_zhe0412@hotmail.com (Z.S.)
3 Key Laboratory of Cosmic Rays, Tibet University, Ministry of Education, Lhasa 850000, China
* Correspondence: wangbin403403@126.com (B.W.); yfchen@uestc.edu.cn (Y.C.); wsf@utibet.edu.cn (S.W.)
† These authors contribute equally to this work.

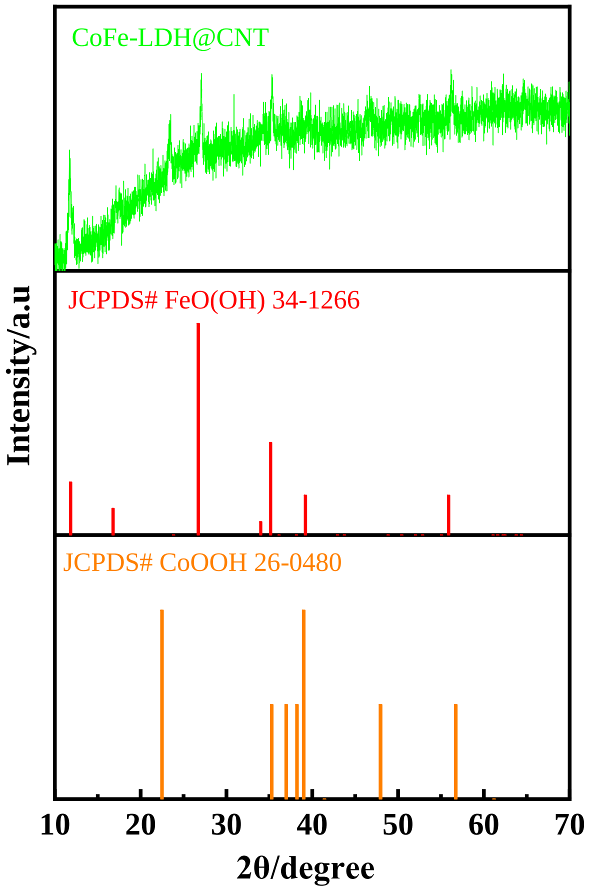

Figure S1. XRD pattern of the CoFe-LDH@CNT precursor.

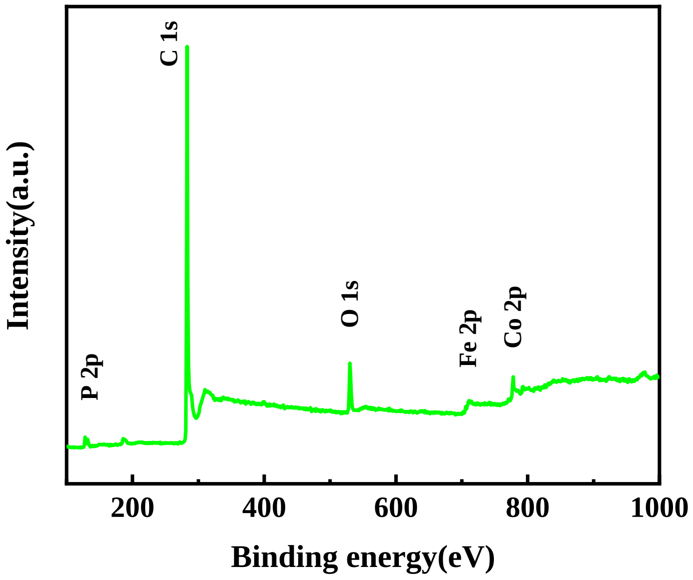

Figure S2. The XPS survey spectrum of $CoP/FeP_4@CNT$.

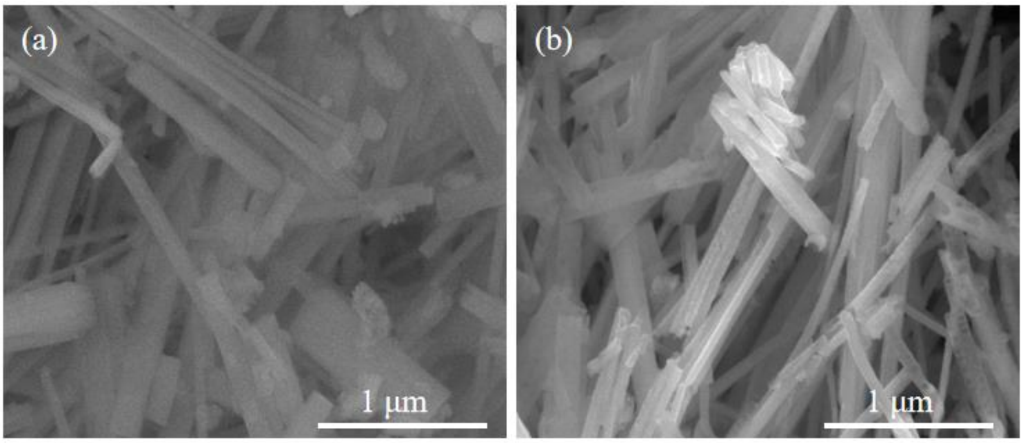

Figure S3. SEM morphology images of (**a**) CoFe-LDH, (**b**) CoFeP.

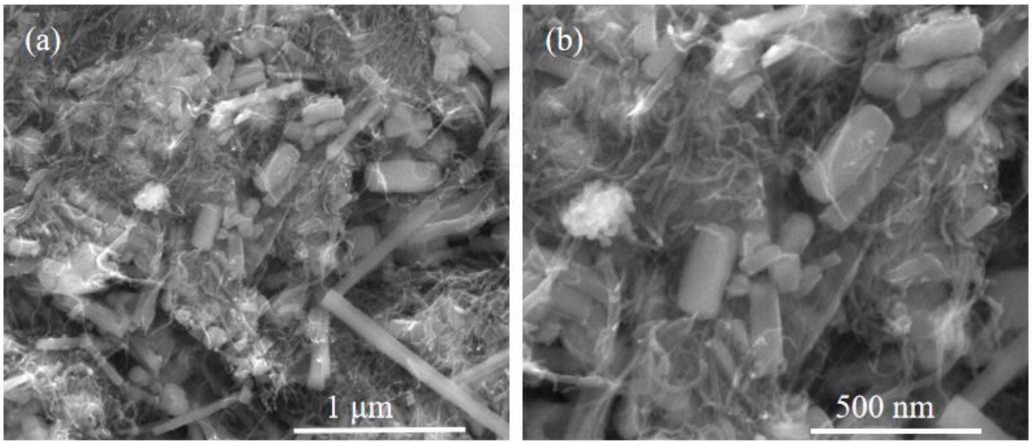

Figure S4. (**a**,**b**)SEM images of CoFe-LDH@CNT from different magnifications.

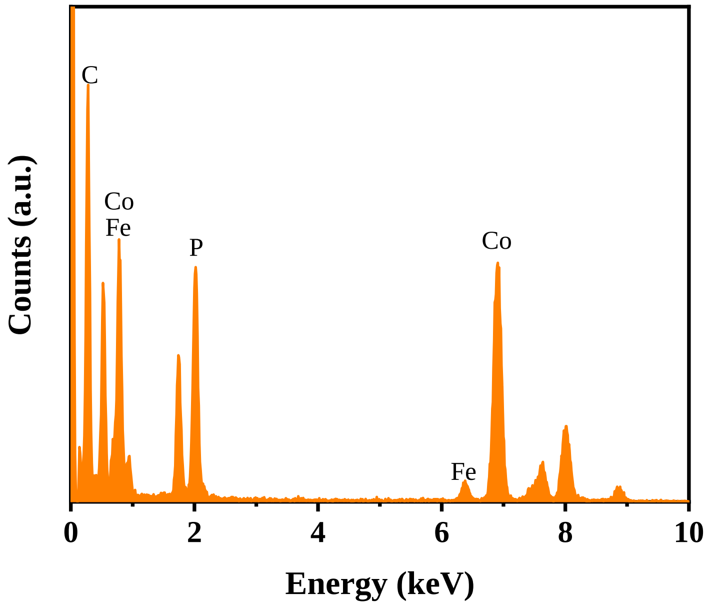

Figure S5. EDX spectrum of CoP/FeP$_4$@CNT.

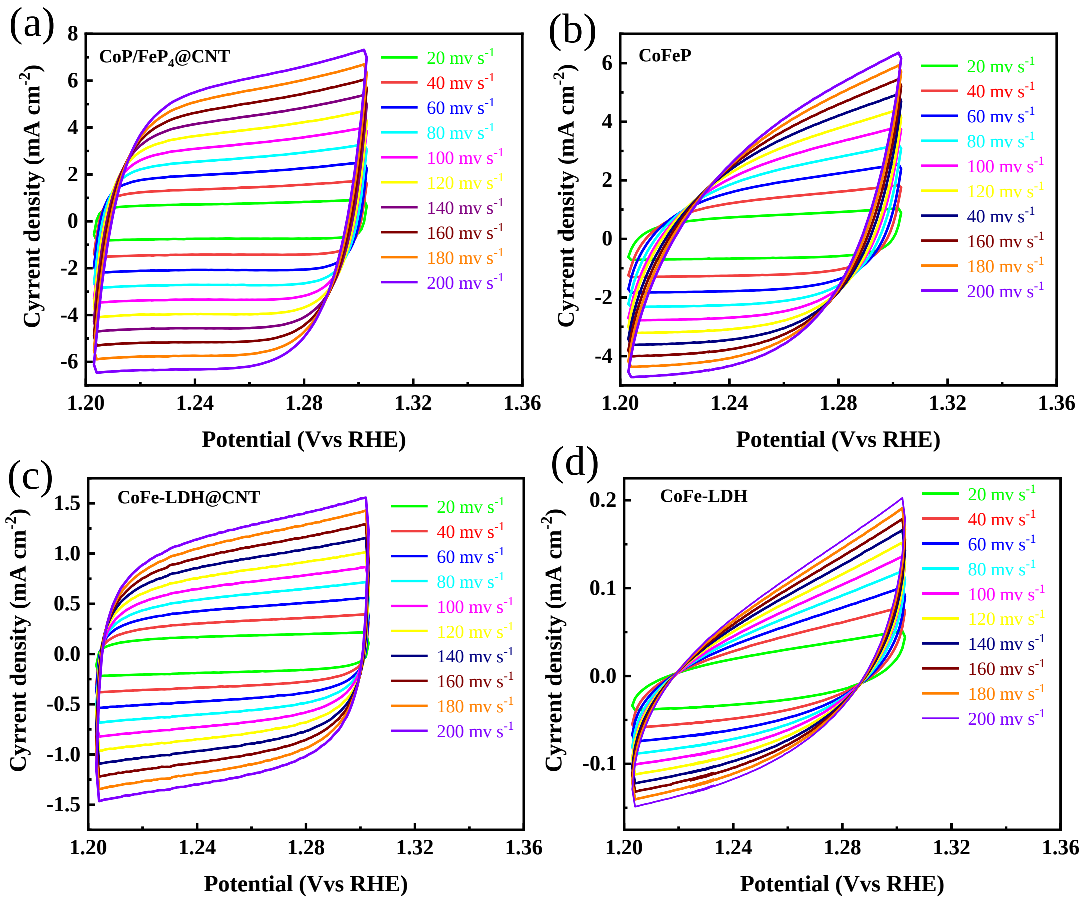

Figure S6. (**a–d**) Voltammograms for the OER in an alkaline electrolyte.

© 2021 by the authors. Licensee MDPI, Basel, Switzerland. This article is an open access article distributed under the terms and conditions of the Creative Commons Attribution (CC BY) license (http://creativecommons.org/licenses/by/4.0/).
